# A Dual GC–MS and LC–MS/MS Analytical Strategy for Evaluating Free Chlorine Toxicity in Aqueous Samples

**DOI:** 10.1002/rcm.70151

**Published:** 2026-07-31

**Authors:** Avital Shifrovitch, Adi Tzadok, Moran Madmon, Avi Weissberg

**Affiliations:** ^1^ Department of Analytical Chemistry Israel Institute for Biological Research (IIBR) Ness Ziona Israel

**Keywords:** acute toxicity, aqueous samples, derivatization, free chlorine, GC–MS, LC–MS/MS

## Abstract

**Rationale:**

Chlorine gas is a highly toxic industrial chemical that poses a significant risk in accidental releases and deliberate terrorist attacks. Reliable analytical methods for the determination of chlorine in aqueous samples are therefore required for forensic and environmental investigations. This study aimed to develop a rapid and selective mass spectrometry‐based method for chlorine determination following derivatization.

**Methods:**

Chlorine in aqueous samples was derivatized with methyl 2‐(3,4,5‐trimethoxyphenyl)acetate (MTMPA) at 50°C for 15 min, yielding methyl 2‐(2‐chloro‐3,4,5‐trimethoxyphenyl)acetate (Cl‐MTMPA). The derivatization product was analyzed by gas chromatography–mass spectrometry (GC–MS) and liquid chromatography–tandem mass spectrometry (LC–MS/MS). While LC–MS/MS analysis was performed directly, GC–MS analysis required an additional liquid–liquid extraction step. Reaction conditions were optimized to maximize derivatization efficiency.

**Results:**

The derivatization reaction provided a high yield of Cl‐MTMPA under the optimized conditions. GC–MS and LC–MS/MS analyses produced a common major fragment ion at *m/z* 215.0. Further structural confirmation by liquid chromatography–high‐resolution tandem mass spectrometry (LC‐HRMS/MS) revealed a characteristic product ion at *m/z* 215.0473 generated from the precursor ion at *m/z* 275.0681. Calibration curves covering acute toxicologically relevant chlorine concentrations (5–200 mg/L) showed excellent linearity, with correlation coefficients (*R* ^2^) exceeding 0.99.

**Conclusions:**

A rapid and selective derivatization‐based method was developed in which the resulting chlorine derivative is readily analyzed by both GC–MS and LC–MS/MS. The method affords reliable analyte identification through characteristic fragmentation and excellent quantitative performance, making it suitable for forensic toxicology and environmental investigations involving chlorine exposure.

## 1 Introduction

Chlorine gas is a toxic substance classified as a pulmonary irritant. Although it is widely used in industrial applications, chlorine has also been used as a chemical warfare agent, notably in World War I, as well as in more recent conflicts such as the Iraq and Syrian Wars, where it caused significant human casualties [1, 2]. Large‐scale industrial production further increases these risks. For example, the United States produces millions of tons of chlorine each year, with substantial quantities stored and transported near populated areas, creating potential safety concerns [3]. Both accidental releases (e.g., industrial incidents) and intentional misuse represent realistic scenarios that could result in contamination of water, beverages, or food [4]. When dissolved in water, chlorine forms hypochlorous acid (HOCl) and hydrochloric acid (HCl). HOCl can further dissociate into hypochlorite ions (OCl^−^), and together these species are referred to as free chlorine. Despite its toxicity, chlorine is widely used for disinfection. It plays an important role in medical and biohazard decontamination [5] and is commonly applied to treat drinking water, greywater, swimming pools, and recycled water systems, where microbial contamination may be present [6]. Maintaining appropriate chlorine levels is essential for effective disinfection and minimizing health risks. In drinking water treatment, residual chlorine is typically maintained below 5 mg/L in the final stages. Exposure to higher concentrations may cause respiratory irritation and has been associated with adverse health effects, including asthma and allergies, and possibly more severe outcomes [7]. Therefore, careful monitoring of free chlorine levels is necessary to ensure public health and environmental safety of water. A recent comprehensive review detailed various analytical methods for detecting chlorine in aqueous and gaseous environments [8]. In addition, a recent review described a variety of chromatographic‐ and mass spectrometry (MS)–based methods for analyzing chlorine‐derived oxidants in seawater [9]. Chlorine detection methods include iodometric titration [10], ion chromatography [11, 12], spectrophotometric, colorimetric, and fluorometric methods [12, 13, 14, 15, 16, 17], chemiluminescence [18, 19], and liquid chromatography (LC) [20, 21]. MS‐based approaches coupled with chromatographic separation, such as gas chromatography (GC) and LC, are attractive alternatives for free chlorine determination because of the high selectivity of MS detection. To date, only a few GC–MS methods [21, 22, 23] and a single LC–MS/MS method [24] have been described for the analysis of free chlorine following chemical derivatization. Generally, each technique offers distinct advantages. LC–MS/MS provides higher sensitivity and requires minimal sample preparation; however, it necessitates the use of an LC‐compatible derivatizing agent. In contrast, GC–MS is operationally simple and robust, making it suitable for on‐site detection and identification, rather than exclusive laboratory use. In this study, we present a novel derivatizing agent that reacts with chlorine to form a product suitable for both GC–MS and LC–MS/MS analysis, allowing the efficient determination of free chlorine in water at toxicologically relevant levels.

## 2 Materials and Methods

### 2.1 Chemicals, Reagents, and Preparation Protocol

#### 2.1.1 Chemicals and Reagents

Commercial Bleach was sourced from Wako Pure Chemical Industries Ltd. (Osaka, Japan). Methyl 2‐(3,4,5‐trimethoxyphenyl) acetate was obtained from Tzamal‐D‐Chem Laboratories Ltd., Petah Tikva, Israel. MS‐grade water, methanol, and ammonium formate were obtained from Biolab (Jerusalem). Methyl tert‐butyl ether (MTBE) was obtained from Sigma–Aldrich (St. Louis, MO, USA). In addition to MS‐grade water, municipal tap water was used.

#### 2.1.2 Derivatization of Free Chlorine for GC–MS and LC–MS/MS Analysis

Diluted bleach (1 mL of a 5–200‐mg/L solution) was mixed with 10 mg of methyl 2‐(3,4,5‐trimethoxyphenyl) acetate in an Eppendorf vial, stirred at 50°C for 15 min, diluted 1:1000 with water and subsequently analyzed using LC–MS/MS. For GC–MS analysis, following derivatization, 1 mL of MTBE was added to the vial. After stirring for 10 s, the upper phase was collected and diluted (1:10, v/v) with MTBE before analysis.

#### 2.1.3 Solutions Preparation

A free chlorine stock solution of 1 mg/mL was prepared from aqueous sodium hypochlorite and standardized by the iodometric method [10]. Working standards (blank, 5, 10, 50, 100, and 200 mg/L) were prepared in triplicate by dilution with water.

### 2.2 Instrumentation

#### 2.2.1 GC‐EI‐MS

Samples were analyzed using an Agilent 7890B GC coupled to a 5977A mass‐selective detector (Agilent Technologies Inc., Wilmington, Delaware, USA). The GC–MS parameters were set according to our previous study [25] and are described below. An aliquot of 4 μL of each sample was introduced into the GC system using a PAL RSI 85 autosampler (CTC Analytics, Zwingen, Switzerland). The GC system was additionally connected in parallel to a nitrogen phosphorus detector (NPD) and a pulsed flame photometric detector (PFPD, OI Analytical, Texas, USA). Chromatographic separation was performed using a DB‐5MS capillary column (15 m × 0.25 mm × 1.00 μm) with helium as the carrier gas. The initial carrier gas flow rate was set to 5 mL/min and maintained for 0.1 min, followed by a reduction to 2 mL/min at a rate of 7.5 mL/min^2^, where it was maintained for 6 min. The flow rate was then increased back to 5 mL/min at a rate of 5 mL/min^2^. Analyses were conducted in splitless injection mode. The injector and transfer line temperatures were maintained at 280°C. The oven temperature was initially set to 70°C, then increased to 315°C at a rate of 40°C/min and held for 3 min. Mass spectra were acquired after a solvent delay of 0.9 min. The mass spectrometer was operated in electron ionization mode at 70 eV, with a mass range of *m/z* 45–495 and an ion source temperature of 230°C. The total run time was 9 min.

#### 2.2.2 LC–ESI–MS/MS (QTRAP)

Chromatographic separation was performed using an Agilent 1290 high‐performance LC (LC system, Palo Alto, CA, USA). MS/MS experiments were conducted using an Applied Biosystems 6500+ quadrupole linear‐ion trap mass spectrometer (QTRAP, AB SCIEX, Framingham, MA, USA) operated with Analyst software (Version 1.7.2) and equipped with a Turbo V ion source in the positive ESI mode. Separation was achieved using a reversed‐phase LC column (Gemini C18, 3.0 μm, 150 × 2.1 mm i.d.; Phenomenex, Switzerland) at a flow rate of 0.3 mL/min. The column temperature was maintained at 40°C throughout the analysis. The mobile phase consisted of solvent A (water containing 1 mM ammonium formate) and solvent B (methanol containing 1‐mM ammonium formate). The gradient program started at 5% solvent B, followed by a linear increase to 90% B over 5 min. The mobile phase composition was then rapidly returned to the initial conditions (5% B) over 0.01 min and maintained for 3 min to allow column re‐equilibration before the next injection. The total chromatographic run time was 11 min, and the injection volume was 5 μL. The ESI inlet conditions were set as described in our previous report [24] and are summarized below. The ionization source parameters were as follows: Gas 1 (air) pressure, 60 psi; Gas 2 (air) pressure, 30 psi; ion spray voltage, 5500 V; ion source temperature, 500°C; and curtain gas (nitrogen) pressure, 35 psi. Enhanced product ion (EPI) experiments were conducted with the collision gas set to the “medium” setting, while collision energies were varied between 10 and 50 V.

#### 2.2.3 LC–ESI–MS/MS (Orbitrap)

Analysis was performed using an Agilent 1290 HPLC system (Palo Alto, CA, USA) coupled to a Q Exactive Plus Orbitrap mass spectrometer (Thermo Fisher Scientific, Bremen, Germany) equipped with a heated electrospray ionization (HESI) source and operated in positive ionization mode. The chromatographic conditions were identical to those used for the QTRAP method described above. The detailed Orbitrap parameters have been previously reported [24] and are illustrated below: The HESI source parameters were set as follows: spray voltage, 1.25 kV; sweep gas, 2 arbitrary units; auxiliary gas heater temperature, 400°C; sheath gas flow rate, 45 arbitrary units; and capillary temperature, 275°C. Collision energies were adjusted within the range of 20–40 V. Initially, all compounds were acquired in full‐scan mode over an *m/z* range of 60–900. Subsequently, data‐independent acquisition (DIA) MS/MS analyses were carried out using collision energies between 10 and 40 V with a normalized collision energy (NCE) value of 35 V.

#### 2.2.4 GC–EI–MS Spectrum of the Monochlorinated Product (Cl‐MTMPA)

Several fragments were observed in the EI–MS spectrum of the monochlorinated product. The molecular ion was observed at *m/z* 274.1, accompanied by a characteristic chlorine isotope peak at *m/z* 276.1, with an intensity approximately three times lower. Additional fragments were detected at *m/z* = 215.0 and 217.0. The derivative was identified in full‐scan mode, and quantification was performed using the extracted ion chromatogram (EIC) at *m/z* 215.0. Low‐abundance fragments were also observed at *m/z* 239.1, 259.1, and 261.1.

#### 2.2.5 LC–ESI–MS/MS Spectrum of the Mono‐Chlorinated Product (Cl‐MTMPA)

Multiple product ions were observed in the ESI–MS/MS spectrum of Cl‐MTMPA, which enabled the establishment of several MRM transitions. Among them, three transitions were selected: *m/z* 275.1 → 215.0 (ce 16 V), *m/z* 277.1 → 217.0 (ce 16 V), and *m/z* 275.1 → 137.0 (ce 43 V), with a declustering potential of 50 V. The transition *m/z* 275.1 → 215.0, which exhibited the highest signal‐to‐noise ratio, was selected as the quantifier, and the remaining transitions were used as qualifiers to enhance the selectivity. The injection volume was 5 μL.

#### 2.2.6 LC–ESI–MS/MS (Orbitrap) Spectrum of cl‐MTMPA

High‐resolution MS (HR‐MS) analysis of Cl‐MTMPA revealed characteristic fragment ions at *m/z* 215.0473, 200.0238, 182.0132, 172.0288, 165.0548, and 137.0598. Tentative fragment structures were assigned based on accurate MS/MS measurements with mass errors below 3 ppm.

## 3 Results and Discussion

In accordance with the World Health Organization (WHO), free chlorine concentrations in water, particularly drinking water, should be regularly monitored to ensure compliance with safety standards and to protect consumers from potential adverse health effects. Recent routine, non‐MS methods for monitoring free chlorine have been reported and are generally classified into conventional, optical, and electrochemical techniques [26]. MS detecting methods, when coupled with GC or LC, offer superior selectivity by reducing matrix interferences and providing structural confirmation based on molecular weight and fragmentation patterns. Additionally, these analytical platforms enable the simultaneous determination of free chlorine and other water quality analytes in a single run. Accordingly, we investigated the feasibility of developing methods based on GC–MS and LC–MS/MS analysis. Our goal was to develop a method for determining free chlorine at concentrations ranging from 5 to 200 ppm, a range that is highly relevant for forensic and environmental safety applications. According to the WHO, 5 ppm represents the upper limit recommended for safe monitoring, whereas concentrations exceeding 5 ppm correspond to levels that may be encountered in hazardous exposure scenarios resulting from accidental release, equipment failures, or intentional contamination.

### 3.1 Designing a Derivatizing Agent Compatible With Both GC–MS and LC–MS/MS Analysis

**FIGURE 1:**
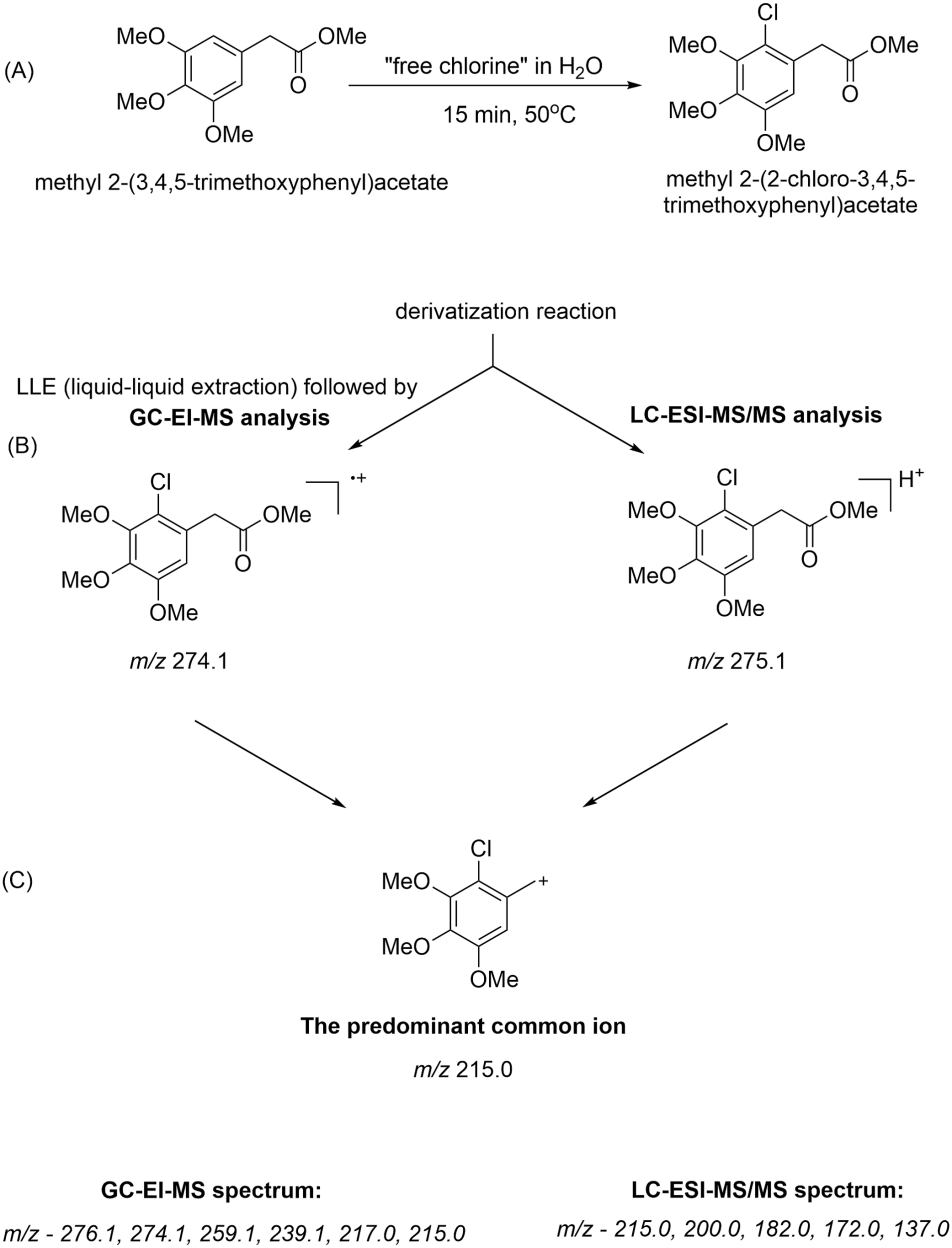
General scheme of the dual analytical approach (GC–MS and LC–MS/MS). (A) Reaction scheme for the formation of the monochlorinated product, methyl 2‐(2‐chloro‐3,4,5‐trimethoxyphenyl) acetate, (B) sample preparation required prior to GC–MS and LC–MS/MS analysis, (C) and major masses observed in the GC–EI–MS/LC–ESI–MS/MS spectra.

Free chlorine species, including Cl_2_, HOCl, and OCl^−^, are not amenable to direct mass spectrometric detection because of their high reactivity and lack of stable ionization behavior. Consequently, chemical derivatization can be employed to transform these species into products that are stable and detectable by MS. One effective strategy involves electrophilic aromatic halogenation, a subclass of electrophilic aromatic substitution reactions. Selecting a derivatizing agent with a benzene ring containing multiple electron‐donating groups, which increase the ring's electron density, is an effective strategy for accelerating the chlorination reaction. Previous research suggests that 1, 3, 5‐trimethoxybenzene (TMB) reacts efficiently with free chlorine in aqueous solutions, and the resulting chlorinated product (Cl‐TMB) can be quantified using GC–MS [21, 23]. However, the resulting derivatization product is incompatible with LC–MS/MS analysis. Moreover, although the TMB derivatization approach enables GC–MS detection of free chlorine, its reported linear dynamic range, up to 120 μM as Cl_2_ (8.51 mg/L), is insufficient for reliable quantification at the elevated concentrations associated with highly toxic conditions. Another derivatization reagent for the detection and identification of free chlorine by GC–MS is styrene, which reacts through electrophilic aromatic substitution. The relatively large amount of styrene required (20 μL) enables quantification of free chlorine concentrations up to 100 mg/L, making this method suitable for monitoring highly toxic chlorine levels. However, similar to TMB, styrene is incompatible with LC–MS, limiting its application in LC–MS‐based analytical methods [22]. The inclusion of a functional group capable of protonation or deprotonation is required for effective LC–MS/MS detection. Additionally, the derivatizing agent should be chosen such that its derivatization product produces a distinctive fragment or product ion from the molecular (precursor). We previously selected the reagent 3,4,5‐trimethoxyphenylacetic acid for LC–MS/MS analysis because of the presence of three electron‐donating methoxy substituents and an acetic acid moiety, which enhance sensitivity in negative ESI–MS/MS [24]. The incorporation of an acetic acid moiety improves MS detection sensitivity; however, it is chromatographically suboptimal, as it often results in reduced retention and broader peaks in reverse‐phase LC analysis. In addition, the acetic acid moiety increases the boiling point of the derivatization product, rendering it unsuitable for GC–MS. Therefore, the commercially available methyl ester derivative of the acetic acid moiety was used to increase volatility and enable GC–MS analysis. This modification simultaneously improved the LC chromatographic performance by enhancing the retention and peak shape while maintaining a proton‐acceptor site for sensitive positive‐ion MS detection. The derivatization reaction and the sample preparation required prior to GC–MS and LC–MS/MS are illustrated in Figure 1A,B, respectively.

### 3.2 Structural Characterization of the Monochlorinated Product by GC–EI–MS and LC–ESI–MS/MS

**FIGURE 2:**
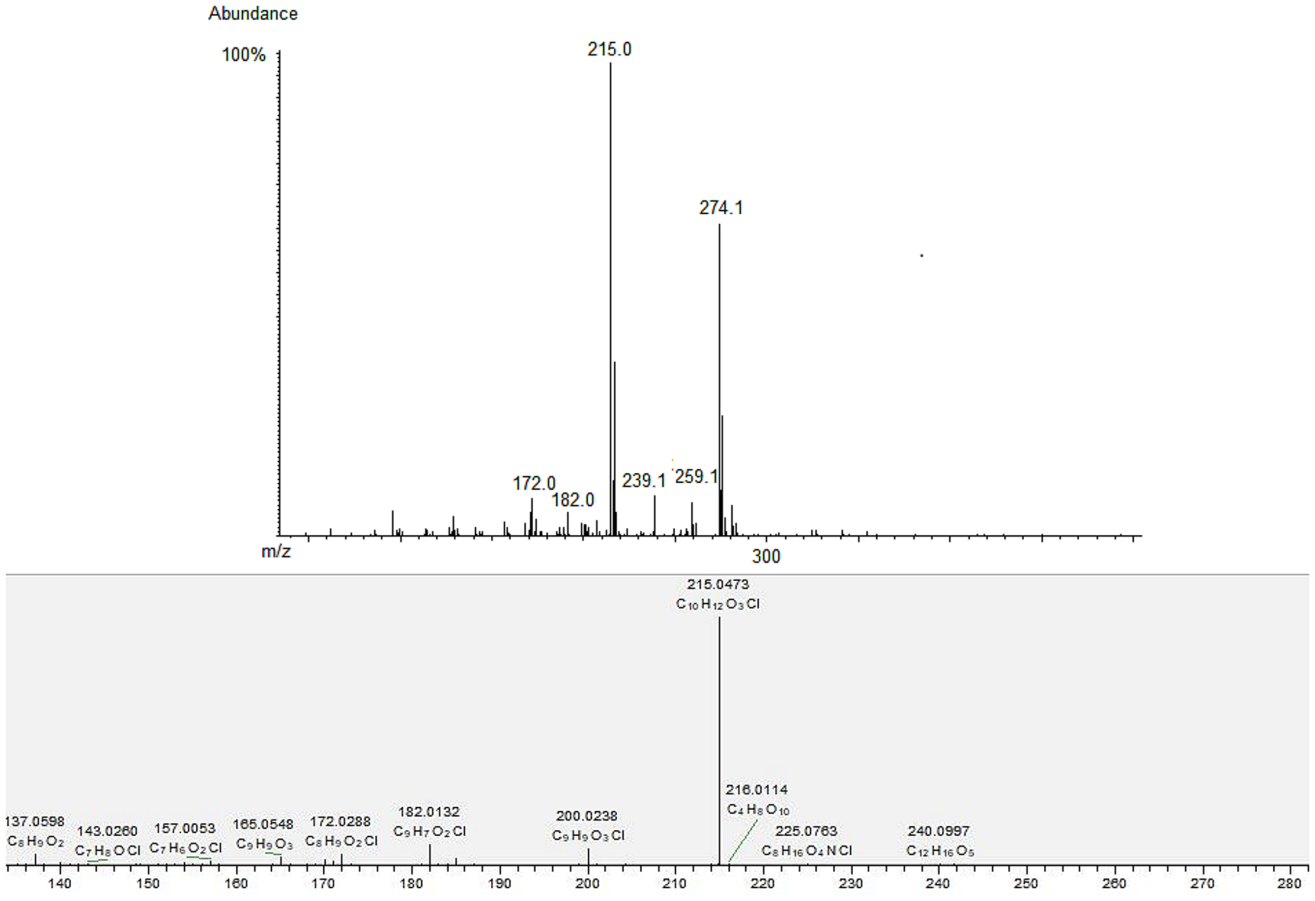
GC–EI–MS spectrum of the monochlorinated product following derivatization and liquid–liquid extraction (upper panel). LC–ESI–MS/MS (Orbitrap) spectrum of the same product at 35‐eV normalized collision energy (lower panel).

**FIGURE 3:**
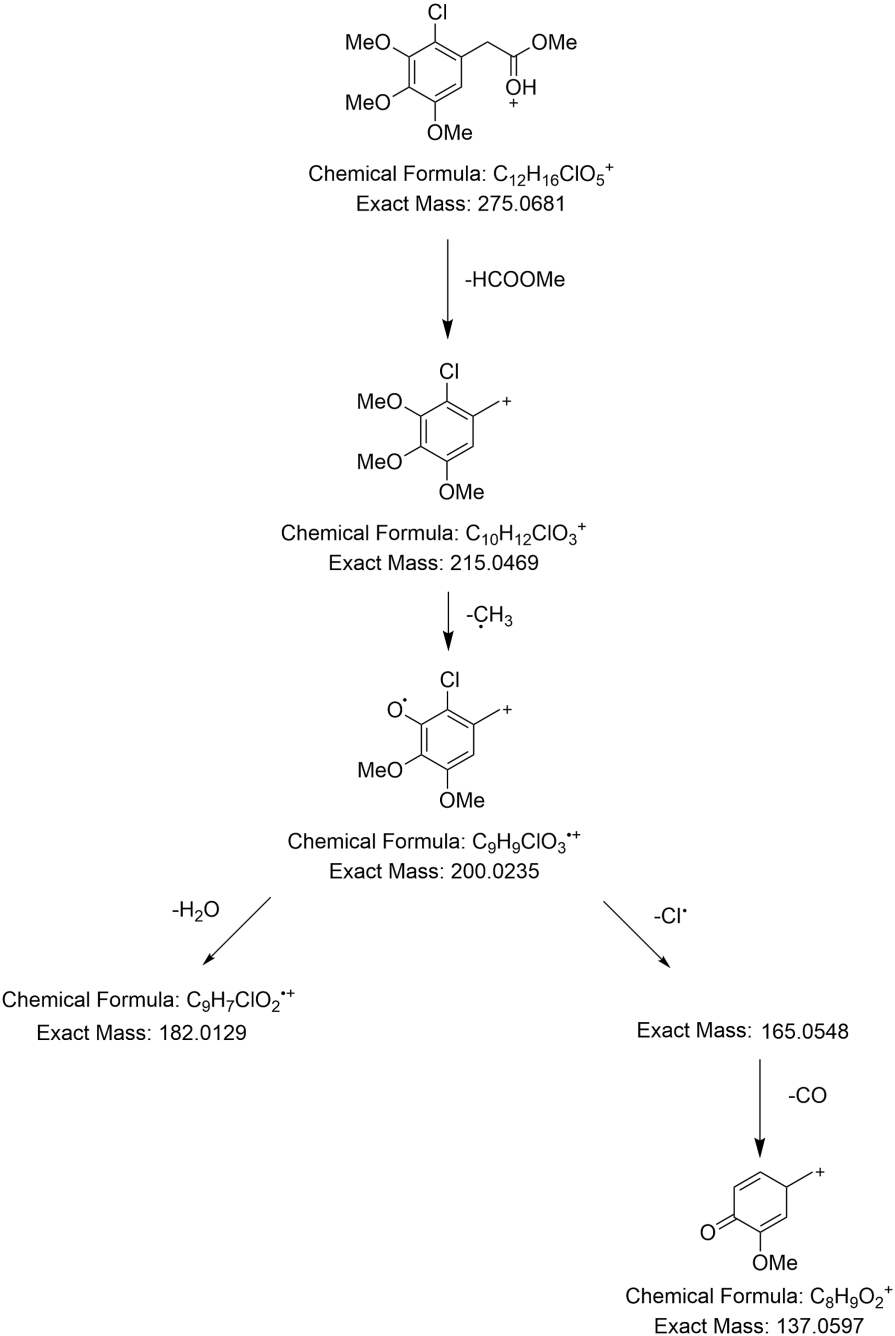
Proposed structures of product ions detected in the LC–ESI–MS/MS spectrum of the monochlorinated product.

In general, several common masses were observed in both mass spectra. The GC–EI–MS spectrum of the derivatization product exhibited a molecular ion at *m/z* 274.1 with a retention time of 5.2 min (Figure 2, upper panel). Owing to the presence of a chlorine atom, an additional isotopic ion at *m/z* 276.1 was observed, corresponding to ^37^Cl and appearing at approximately one‐third the intensity of the ion at *m/z* 274.1. Additionally, low‐abundance fragments at *m/z* 259.1 and 261.1 were observed, corresponding to the loss of a methyl radical, while a fragment at *m/z* 239.1 resulted from the loss of a chlorine radical. Two major fragments appeared at *m/z* 215.0 and 217.0, with an intensity ratio of 3:1, consistent with the chlorine isotopes. These fragments were attributed to the formation of the most stable benzyl cation/tropylium ion (Figure 1C). Minor fragments at *m/z* 182.0 and 172.0 were also observed. In addition, Orbitrap–ESI–MS/MS experiments were performed on the monochlorinated product (Cl‐MTMPA) using collision energies of 10–50 eV. The Orbitrap ESI–MS/MS spectrum exhibited characteristic product ions at *m/z* 215.0473, 200.0238, 182.0132, 172.0288, 165.0548, and 137.0598, corresponding to calculated exact masses of 215.0469, 200.0235, 182.0129, 172.0291, 165.0548, and 137.0597, respectively. The measured masses agreed well with the theoretical values, with mass errors of +1.86, +1.50, +1.65, −1.74, 0.00, and +0.73 ppm, respectively (Figure 2, lower panel). The proposed structures of these ions are presented in Figure 3. The *m/z* 215.0473 ion, assigned to a benzylic cation/tropylium structure, was generated via the elimination of methyl formate from the [M + H]^+^ precursor.

The fragment ion at *m/z* 200.0238 results from the combined loss of a methyl formate molecule and a methyl radical. The ion at *m/z* 182.0132 likely formed through water loss from *m/z* 200.0238. The ion at *m/z* 165.0548 was possibly produced via the loss of a chlorine radical from *m/z* 200.0238, while the ion at *m/z* 137.0597 likely arose from the loss of CO from *m/z* 165.0548.

Three MRM transitions of the monochlorinated derivatization product (275.1 → 215.0, 277.1 → 217.0 for the chlorine isotope, and 275.1 → 137.0) were acquired using QTRAP. These transitions were selected for detection and confirmation, exhibiting signal intensity ratios of ~33:11:2 and a consistent retention time (RT ≈ 6.8 min).

### 3.3 Assessing the Reactivity of Methyl 2‐(3,4,5‐Trimethoxyphenyl)acetate (MTMPA) Toward Free Chlorine Using LC–MS/MS

In our previous study, we developed an LC–MS/MS method with a linear dynamic range of 0.1–10 μg/mL using a derivatization reagent concentration of 1 mg/mL [24], suitable for the routine determination of free chlorine in drinking water. In the present study, we aimed to develop a derivatization method compatible with both GC–MS and LC–MS/MS for quantifying higher free chlorine concentrations (5–200 μg/mL), exceeding both the analytical range of the previous method and the WHO guideline value for chlorine in drinking water (> 5 μg/mL). To achieve these higher concentration levels, the derivatization reagent concentration was increased tenfold, from 1 to 10 mg/mL. High derivatizing agent concentrations (1 and 10 mg/mL) were tested to achieve efficient derivatization at elevated free chlorine levels (up to 200 μg/mL). The reaction times of 5, 15, 30, and 60 min were evaluated at both ambient temperature and 50°C. Maximal signal intensity was achieved after 15 min at 50°C using a derivatizing agent concentration of 10 mg/mL. Reducing the concentration of the derivatizing agent by an order of magnitude led to a significant reduction in the MRM signal intensity of the derivatization product. Reactions performed at ambient temperature yielded lower signal intensities than those at 50°C. Additionally, a reaction time of 15 min was sufficient to achieve quantitative conversion. Consequently, the optimized derivatization conditions consisted of incubating 10 mg of the derivatizing agent with 1 mL of the aqueous sample in an Eppendorf tube, followed by shaking for 15 min at 50°C and dilution prior to LC–ESI–MS/MS analysis.

### 3.4 Method Performance Assessment

At doses of 1–4 mg/L, free halogens can inactivate pathogens in drinking water, wastewater, and recreational water. The WHO recommends an upper limit of approximately 5 mg/L for free chlorine in drinking water, 5 mg/L for swimming pools, and 10 mg/L for spa waters, as higher concentrations may be harmful to health [7]. Free chlorine concentrations in the tens to hundreds of mg/L far exceed normal drinking‐water residuals and may indicate hazardous ingestion exposure, potentially resulting from accidents, equipment failures, or intentional contamination.

Therefore, monitoring is essential for public health protection and the detection of chemical hazards. Accordingly, GC–MS and LC–MS/MS methods were validated using tap water spiked at relevant concentration levels (5–200 mg/L). The method performance was assessed in terms of linearity, repeatability (intraday precision), limit of identification (LOI), and limit of quantification (LOQ) for free chlorine following its conversion to the monochlorinated product methyl 2‐(2‐chloro‐3,4,5‐trimethoxyphenyl) acetate. For LC–MS/MS analysis, samples were diluted 1:1000 with water prior to injection, whereas for GC–MS analysis, liquid–liquid extraction (> 90% recovery) was performed, followed by a 1:10 dilution with MTBE prior to analysis.

In LC–MS/MS analyses, matrix‐related effects, such as ionization suppression, reduced derivatization efficiency, and product instability, are commonly encountered and are the major causes of signal attenuation. Matrix effects were evaluated by comparing recoveries obtained from MS‐grade water and tap water samples spiked with bleach at concentrations of 5, 10, 50, 100, and 200 mg/L.

**FIGURE 4:**
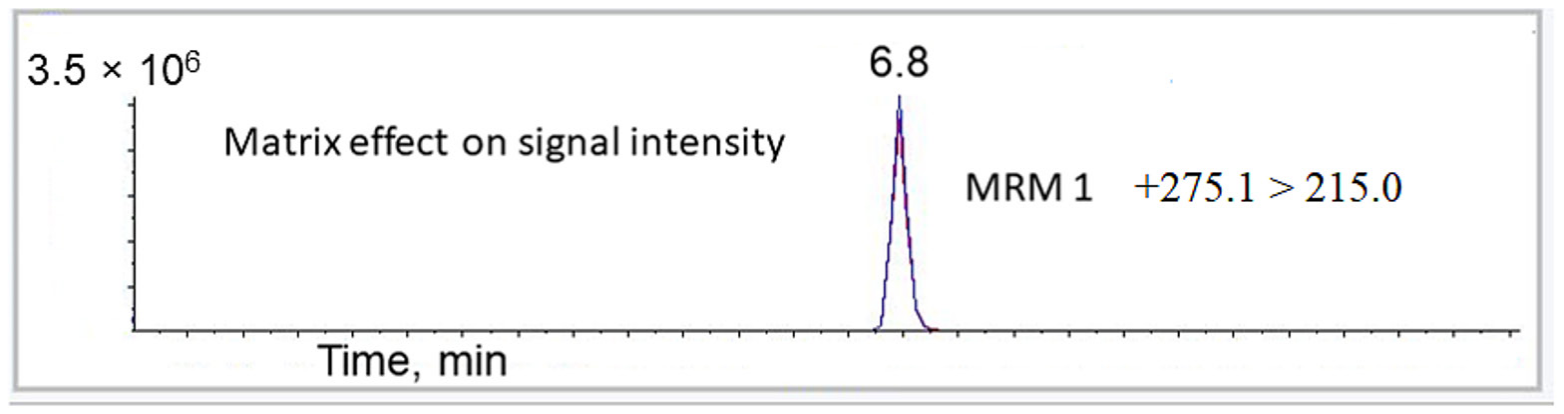
EICs of the major MRM transition (*m/z* 275.1 → 215.0) of the monochlorinated product, methyl 2‐(2‐chloro‐3,4,5‐trimethoxyphenyl) acetate (RT = 6.8 min) in MS‐grade water (red) and tap water (blue). The sample was enriched to a concentration of 50 mg/L in an Eppendorf vial, followed by a 15‐min derivatization reaction at 50°C and dilution prior to LC–MS/MS analysis.

The developed method allows for the assessment of the toxicological relevance of measured exposure concentrations. All samples were analyzed by LC–MS/MS after derivatization with MTMPA. The matrix effects were further assessed by comparing the signal intensity of the most intense MRM transition (*m/z* 275.1 → 215.0) of the monochlorinated derivatization product in tap water with that observed in MS‐grade water. The results indicated limited matrix‐induced signal suppression or enhancement. A representative comparison of the signal intensities at 50 mg/L is presented in Figure 4.

**FIGURE 5:**
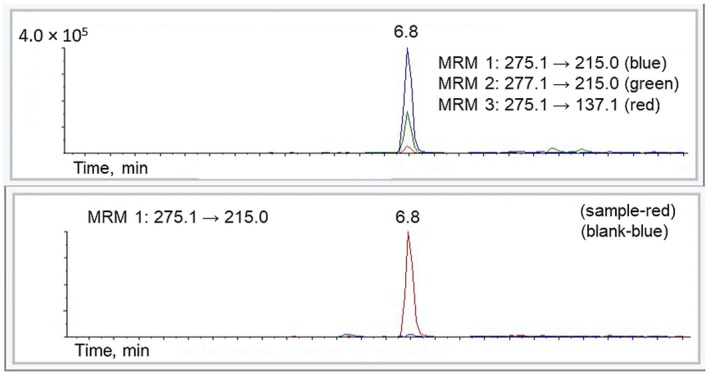
LC–MS/MS analysis showing extracted ion chromatograms (EICs) of the three main MRM transitions (*m/z* 275.1 → 215.0, 277.1 → 217.0, and 275.1 → 137.1) for the monochlorinated product Cl‐MTMPA (5 mg/L, LOQ level, RT = 6.8 min; upper chromatogram). Overlay chromatograms of the sample spiked at 5 mg/L (LOQ level, S/*N* ≥ 10; red) and blank following derivatization are shown (lower chromatogram).

**FIGURE 6:**
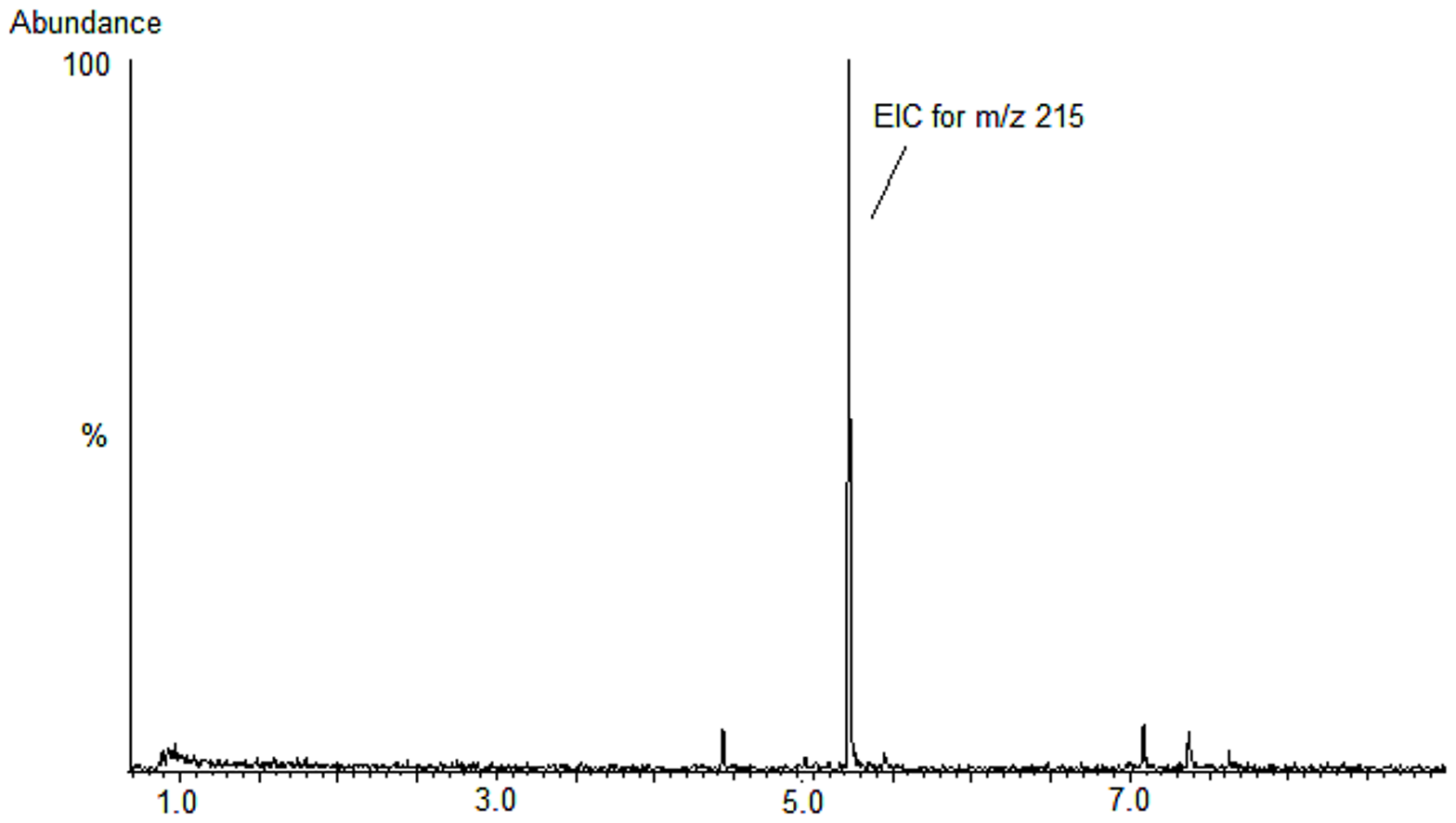
GC–EI–MS analysis displayed the EIC of *m/z* 215.1 for a concentration of 5 mg/L, at the LOQ level.

The calibration curve for the monochlorinated product, methyl 2‐(2‐chloro‐3,4,5‐trimethoxyphenyl) acetate, in water was linear over the range of 5–200 mg/L, with correlation coefficients (*R* ^2^) greater than 0.99 (Figure S1). Reproducibility was assessed in triplicate using samples spiked with 5–200 mg/L. The relative standard deviations (RSDs) for the intraday repeatability of methyl 2‐(2‐chloro‐3,4,5‐trimethoxyphenyl) acetate were below 10%. The LOI and LOQ were established as the lowest concentration of methyl 2‐(2‐chloro‐3,4,5‐trimethoxyphenyl) acetate with chromatographic signals exceeding the peak‐to‐peak background noise by factors of 3 and 10, respectively. The limit of quantitation (S/*N* ≥ 10) was 5 mg/L. The LOI was also 5 mg/L (S/*N* ≥ 3), where three distinct MRM transitions could be detected, and the signal intensity ratio of approximately 33:11:2 was maintained. The validation parameters are listed in Table 1. The stability of methyl 2‐(2‐chloro‐3,4,5‐trimethoxyphenyl) acetate was assessed, and no detectable degradation was observed within 1 day in the reaction mixture. The MRM quantifier transition (*m/z* 275.1 → 215.0) and the two qualifier transitions (*m/z* 277.1 → 217.0 and 275.1 → 137.1) for free chlorine converted to methyl 2‐(2‐chloro‐3,4,5‐trimethoxyphenyl) acetate in tap water at 5 mg/L (LOI) were compared with blank samples, as illustrated in Figure 5. The MRM peaks exhibited no background signals, and the chromatograms were primarily governed by methyl 2‐(2‐chloro‐3,4,5‐trimethoxyphenyl) acetate. In addition, the analytical method was validated using GC–MS in accordance with established validation guidelines. After the derivatization reaction, MTBE was added to the mixture and the upper layer was analyzed by GC–MS. Method performance was evaluated in terms of linearity, sensitivity, and precision. Calibration curves were constructed over the relevant concentration range (5–200 mg/L), demonstrating good linearity with correlation coefficients (*R* ^2^ > 0.99) exceeding accepted criteria (Figure S1). Reproducibility was evaluated in triplicate using samples spiked at concentrations ranging from 5 to 200 mg/L. The relative standard deviations (RSDs) for intraday repeatability of methyl 2‐(2‐chloro‐3,4,5‐trimethoxyphenyl) acetate were below 12%. EIC chromatogram of sample spiked at 5 mg/L (LOQ level, S/*N* ≥ 10; red) following derivatization and analysis are shown in Figure 6. The validation parameters are listed in Table 1. The robustness of the method was further confirmed by consistent performance across multiple analyses, supporting its suitability for reliable quantitative analysis. The LOQ, defined at a signal‐to‐noise ratio of 10, was 5 mg/L. No degradation of the monochlorinated product was observed after 24 h of storage in MTBE at room temperature, indicating its high stability.

**TABLE 1:** Determination of free chlorine: Linear dynamic range, *R* ^2^, LOI, LOQ, and precision of GC–MS and LC–MS/MS methods.

Analytical method	Calibration points (mg/L)	RSD (%)	*R* ^2^	LOI/LOD (mg/L)
GC–MS	5, 10, 50, 100, 200	10, 6, 8, 12, 9	> 0.99	5
LC–MS	5, 10, 50, 100, 200	7, 3, 9, 7, 10	> 0.99	5

*Note:* LC–MS/MS quantification was performed using the MRM transition 275.1 → 215.0, while GC–MS quantification was based on the fragment at *m/z* 215.0.

## 4 Summary and Conclusions

In this study, we developed two analytical methods capable of detecting free chlorine that are appropriate for evaluating acute toxicity and emergency contamination scenarios, such as industrial accidents and intentional water supply poisoning. These methods are based on selective derivatization of free chlorine. The derivatization product, methyl 2‐(2‐chloro‐3,4,5‐trimethoxyphenyl) acetate (Cl‐MTMPA), is compatible with GC–MS and LC–MS/MS and allows the detection and confirmation of free chlorine in aqueous solutions at concentrations relevant to forensic analysis. Sample preparation is rapid and involves the addition of methyl 2‐(3,4,5‐trimethoxyphenyl) acetate to aqueous solutions containing free chlorine. The resulting chlorine derivative exhibited high stability and produced structurally informative fragments and product ions in the EI–MS and ESI–MS/MS spectra, respectively, enabling compound confirmation with a high level of confidence. This analytical approach also allows for the evaluation of whether the measured exposure concentrations are toxicologically relevant to humans.

## Author Contributions

**Avital Shifrovitch:** data curation, investigation, validation, formal analysis, writing – original draft. **Adi Tzadok:** data curation, investigation, validation, formal analysis, writing – original draft. **Moran Madmon:** investigation. **Avi Weissberg:** conceptualization, methodology, supervision, visualization, writing – original draft, writing – review and editing.

## Funding

The authors have nothing to report.

## Conflicts of Interest

The authors declare no conflicts of interest.

## Supporting information


**Figure S1:** Linear dynamic range of the derivatization product (Cl‐MTMPA) in water. The indicated concentration is an average of spiked bleach in triplicate at 5, 10, 50, 100 and 200 mg/L, followed by sample preparation and LC–ESI–MS/MS and GC–MS analyses.

## Data Availability

The data presented in this study, supporting the results, are available in the main text and the Supporting Information. Additional data are available upon request from the corresponding author.
