# Supplementary material for: A Dual GC–MS and LC–MS/MS Analytical Strategy for Evaluating Free Chlorine Toxicity in Aqueous Samples
Source: Rapid Commun Mass Spectrom. 2026 Jul 31;40(20):e70151. doi: 10.1002/rcm.70151 (PMC13425256; doi:10.1002/rcm.70151)
Supplement: Supplementary file 1 [file RCM-40-e70151-s001.docx]

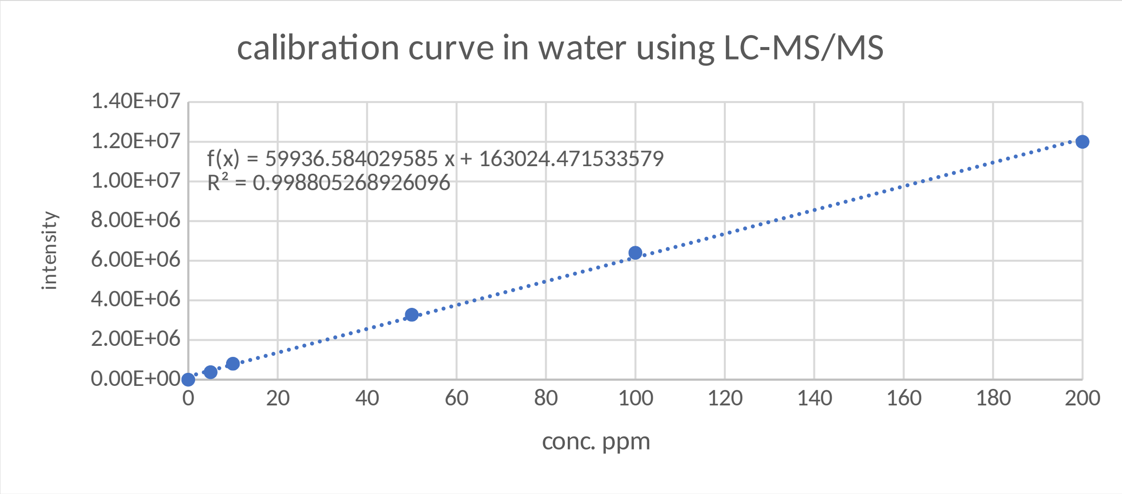
calibration curve in water using LC-MS/MS
f(x) = 59936.584029585 x + 163024.471533579
R² = 0.998805268926096
intensity
1.40E+07
1.20E+07
1.00E+07
8.00E+06
6.00E+06
4.00E+06
2.00E+06
0.00E+00
0
20
40
60
80
100
120
140
160
180
200
conc. ppm

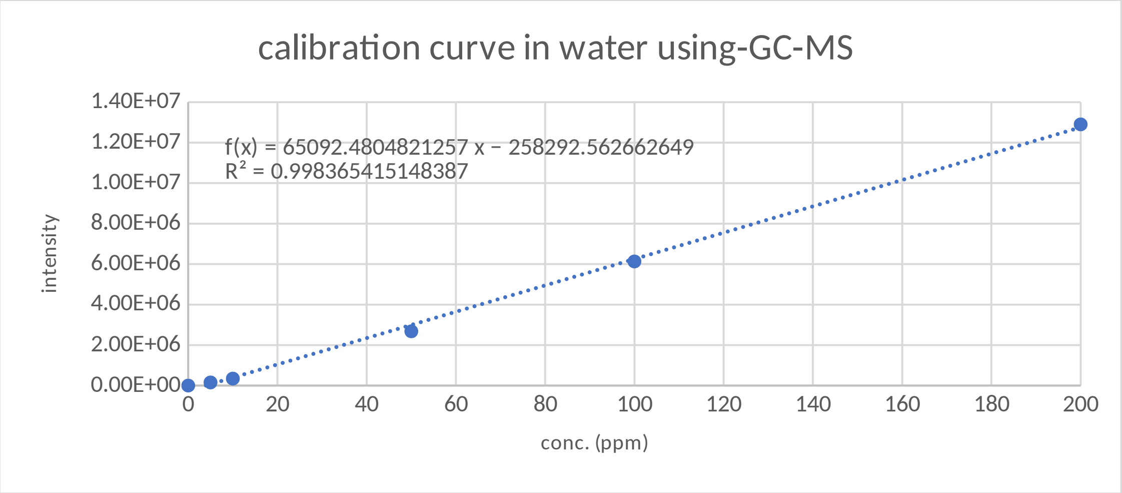
calibration curve in water using-GC-MS
f(x) = 65092.4804821257 x − 258292.562662649
R² = 0.998365415148387
intensity
1.40E+07
1.20E+07
1.00E+07
8.00E+06
6.00E+06
4.00E+06
2.00E+06
0.00E+00
0
20
40
60
80
100
120
140
160
180
200
conc. (ppm)
